# Integrating Calcium Into Antenatal Iron-Folic Acid Supplementation in Ethiopia: Women’s Experiences, Perceptions of Acceptability, and Strategies to Support Calcium Supplement Adherence

**DOI:** 10.9745/GHSP-D-20-00008

**Published:** 2020-09-30

**Authors:** Gina C. Klemm, Zewdie Birhanu, Stephanie E. Ortolano, Yohannes Kebede, Stephanie L. Martin, Girma Mamo, Katherine L. Dickin

**Affiliations:** a Program in International Nutrition, Division of Nutritional Sciences, Cornell University, Ithaca, NY, USA.; b Faculty of Public Health, Department of Health, Behavior and Society, Jimma University, Jimma, Ethiopia.; c Ethiopia–Canada Cooperation Office, Nutrition International, Addis Ababa, Ethiopia.

## Abstract

Recommendations for antenatal calcium supplementation to prevent preeclampsia could substantially reduce maternal mortality, but adherence to multiple daily doses may constrain effectiveness. World Health Organization guidelines recommend 3 daily calcium supplements (1.5–2 g/d), taken separately from 1 iron-folic acid (IFA) supplement; however, limited data suggest lower calcium doses may also be effective. We conducted mixed-methods household trials to identify strategies for supporting adherence and integrating calcium into antenatal IFA supplementation programming in Ethiopia. Participants were randomly assigned to 3 regimens varying in dose and timing and were later given a choice of regimens. Semistructured interviews conducted over 6 weeks explored acceptability, barriers, and facilitators and offered opportunities to choose calcium pill type. Interviews were transcribed, translated, and analyzed thematically. Calcium adherence was measured using medication event monitoring. All participants (N=48) agreed to try supplementation. Adherence barriers included forgetting to take pills when busy or travelling and perceived side effects. Midday doses were the most challenging because of farming, market, and social events; women avoided taking supplements in public due to fear of being perceived as HIV positive. Social support from families, visual reminders, and anticipated benefits motivated adherence. More participants (75%) selected chewable versus conventional supplements due to organoleptic properties, but this preference declined over time. Adherence rates did not substantially differ across regimens with 2 (81.1%), 3 (83.4%), or 4 (77.1%) pill-taking events. Women indicated that the 2-event regimen was more acceptable than 3- and 4-event regimens, but this acceptability was not associated with higher adherence. Consequently, mean daily calcium consumption (811.3 mg) was lower than for 3-event (1,251.1 mg) and 4-event (1,156.4 mg) regimens. Integrating calcium into antenatal IFA supplementation is acceptable to Ethiopian women, with a 3-event regimen yielding the highest consumption rates. Despite women experiencing challenges with midday dosing and stigma, using simple home-based strategies and being counseled on the purpose of supplementation were more effective than reducing dosage for mitigating barriers and improving adherence.

## Abstract (precis)

In household trials of improved practices, rural Ethiopian women were motivated to adhere to antenatal calcium supplementation regimens, and tailored home-based strategies helped them overcome barriers such as regimen complexity, forgetfulness, side effects, and discouragement from others.

## Teaser

**Key Findings:**

- In a small acceptability trial of global antenatal calcium supplementation guidelines, women preferred 2-event regimens to 3- or 4-event regimens, but acceptability was not associated with higher adherence.
- Rural Ethiopian women were motivated and adhered to antenatal micronutrient supplementation despite regimen complexity, forgetfulness, side effects, and discouragement from others.
- Simple home-based strategies and family support contributed to high adherence.

**Key Implications:**

- Formative research can guide antenatal micronutrient supplementation programs by identifying adherence strategies that reflect women’s views and address misperceptions.
- With appropriate program design, supportive counseling, and sufficient access to supplements, women can be highly motivated to adhere to antenatal micronutrient supplementation.

## INTRODUCTION

Antenatal micronutrient supplementation is a cost-effective, scalable approach that can contribute to addressing the persistent challenge of maternal undernutrition^1^^–^^3^; effective and broad implementation of interventions with proven efficacy is urgently needed.^4^^,^^5^

Global guidelines on antenatal calcium supplementation to prevent preeclampsia, a leading cause of maternal mortality, have yet to be widely adopted into national policies.^6^^,^^7^ A systematic review of efficacy trials found that calcium supplementation in pregnancy reduced the risk of preeclampsia by half.^8^ Such trials critically demonstrate the potential impact of micronutrient supplementation, but they provide limited guidance for programming in different settings.^9^

To prevent preeclampsia, the World Health Organization (WHO) suggests 1.5–2 g of calcium per day, taken in 3 divided doses with food and separately from 1 daily iron-folic acid (IFA) supplement.^7^ IFA supplementation to prevent anemia is a longstanding example of a global guideline that has been translated into nutrition policies and programs in many country contexts.^10^ Intake of antenatal IFA is often inadequate in Africa and Asia due to problems of limited coverage and supply, as well as barriers to adherence.^11^ Rates of IFA consumption vary substantially across countries in sub-Saharan Africa, with adherence related to income, education, and initiation and frequency of antenatal care (ANC).^12^ Pooled national antenatal IFA consumption rates in Ethiopia are less than 50%, with much lower rates in rural areas; persistent adherence barriers include lack of first trimester ANC use, inadequate counseling, limited knowledge of supplements and anemia, fear of side effects, forgetfulness, and low adherence support.^13^^–^^15^ Although some reports define “adherence” as consumption of IFA supplements for ≥90 days during pregnancy, it is important to recognize that consumption may be limited by systemic supply constraints (including costs of national procurement and distribution) on women’s access to supplements.^11^^,^^16^^,^^17^

A regimen including calcium as well as IFA is likely to exacerbate implementation and adherence challenges well known within IFA programs.^11^^,^^16^^,^^17^ Research is needed on how best to integrate calcium supplementation into IFA and ANC programming, given that strong supplementation programs are still lacking in many contexts despite decades of IFA advocacy and experience.^18^^–^^20^

In related formative studies in Kenya and Ethiopia, researchers explored how lessons from IFA supplementation can inform calcium initiatives and reduce well-known barriers.^21^^,^^22^ Addi-tional research has examined the influence of regimen complexity or product type on adherence to calcium^23^ and IFA,^24^ as well as the feasibility of calcium supplementation delivery through the health system.^25^^–^^27^ Evidence on acceptability of products, regimens, and counseling messages can inform programs integrating calcium and IFA supplementation and help to maximize utilization.

We aimed to assess factors that influence acceptability and adherence to calcium supplementation among pregnant women in Ethiopia, including supplement type, regimen complexity, administration with IFA, and emergent barriers and facilitators. To assess the acceptability of integrating calcium and IFA supplementation, we used trials of improved practices (TIPs), a mixed-methods approach that tests proposed health interventions and behavioral recommendations to ensure that intervention design is locally acceptable.^28^^,^^29^ During multiple home visits, interviewers and participants discuss the latter’s willingness to try new behaviors, what is difficult or easy about executing selected behaviors, and modifications to improve acceptability.

We explored acceptability and adherence to prenatal calcium supplementation integrated with IFA, using mixed-methods household trials in Ethiopia to inform low-cost strategies for ANC programs.

In addition to qualitatively exploring the motivations, preferences, barriers, and facilitators that influenced acceptability of calcium supplementation, we compared women’s adherence across 3 possible dosing regimens. Previous research found that the number of prescribed daily doses and the regimen complexity were inversely related to adherence of oral medication regimens.^30^^–^^33^ We hypothesized that the regimen suggested by WHO (3 divided doses daily and separate administration of IFA) could result in low adherence rates. In light of evidence that any negative effects of calcium on iron absorption are short-term and calcium supplementation does not adversely affect iron status over time,^34^ we tested a regimen that permitted calcium and IFA to be taken together. Additionally, because evidence suggests that lower calcium doses may be effective,^8^^,^^35^^,^^36^ we tested an alternative lower-dose regimen (1 g/d). We hypothesized that women might find these alternative regimens with fewer daily administrations more acceptable, resulting in higher adherence rates and comparable consumption to the WHO recommended regimen (1.5–2 g/d).

We analyzed women’s perceptions and experiences, as well as adherence rates, to inform policy and program recommendations for translating global guidelines on antenatal calcium supplementation into ongoing ANC programs within Ethiopia and internationally.

We investigated whether simplified prenatal calcium supplementation regimens would be more acceptable than a complex regimen and result in adequate consumption.

## METHODS

### Design

This mixed-methods study used the TIPs approach to conduct a household-level exploration of women’s perceptions and experiences with a new recommended behavior—consumption of antenatal calcium supplementation in addition to IFA. During home visits, women were provided with supplements, counseled on their use, and then asked to participate in a series of in-depth interviews on their experiences and views of acceptability.

In addition, consumption of calcium supplements was assessed quantitatively to triangulate with women’s perceptions and reports and to compare rates of adherence and consumption across 3 regimens. We randomly assigned women to 3 regimens (Table 1) that integrated calcium into the IFA supplementation regimen recommended in Ethiopia.^37^ These represented the calcium regimen proposed in the WHO guidelines (4-event; 1.5 g of calcium), as well as a simplified regimen allowing co-administration of calcium and IFA (3-event; 1.5 g of calcium) and a lower-dose regimen allowing co-administration and reducing calcium dosage (2-event; 1 g of calcium).

**TABLE 1.** Integrated Antenatal Calcium and Iron-Folic Acid Dosing Regimens Assessed in Household Trials in 2 Rural Districts, Ethiopia (N=48)

	**Calcium Supplement** ^a^		**Iron-Folic Acid Supplement** ^b^	
Regimen	Pills per Day (Total Daily Dose)	**Dosing** **Schedule**	**Pill per Day**	**Dosing Schedule**
4-event^c^	3 (1,500 mg)	Morning, midday, evening (with meals)	1	Before bed (separate from calcium)
3-event	3 (1,500 mg)	Morning, midday, evening (with meals)	1	Evening (with calcium)
2-event	2 (1,000 mg)	Morning, evening (with meals)	1	Evening (with calcium)

a 500 mg elemental calcium.

b 65 mg elemental iron and 0.4 mg folic acid.

c Event is the number of discrete times per day that the regimen prescribes taking any supplement.

### Setting and Participants

This study was conducted in 2 rural districts in Oromia region, Ethiopia, from December 2014 to March 2015. In this area, most women (81%) deliver at home, half do not receive ANC, less than one-third (30%) take any iron tablets, and only 35% are informed of pregnancy danger signs.^38^ Limited awareness and minimal diagnostic tools and resources for preeclampsia/eclampsia suggest that many women do not receive adequate care.^21^^,^^39^^,^^40^ To assess the acceptability of calcium in the context of functioning IFA supplementation, we selected rural communities where a recent program, implemented by Nutrition International, had strengthened ANC and IFA supplementation.^41^

**Figure:**
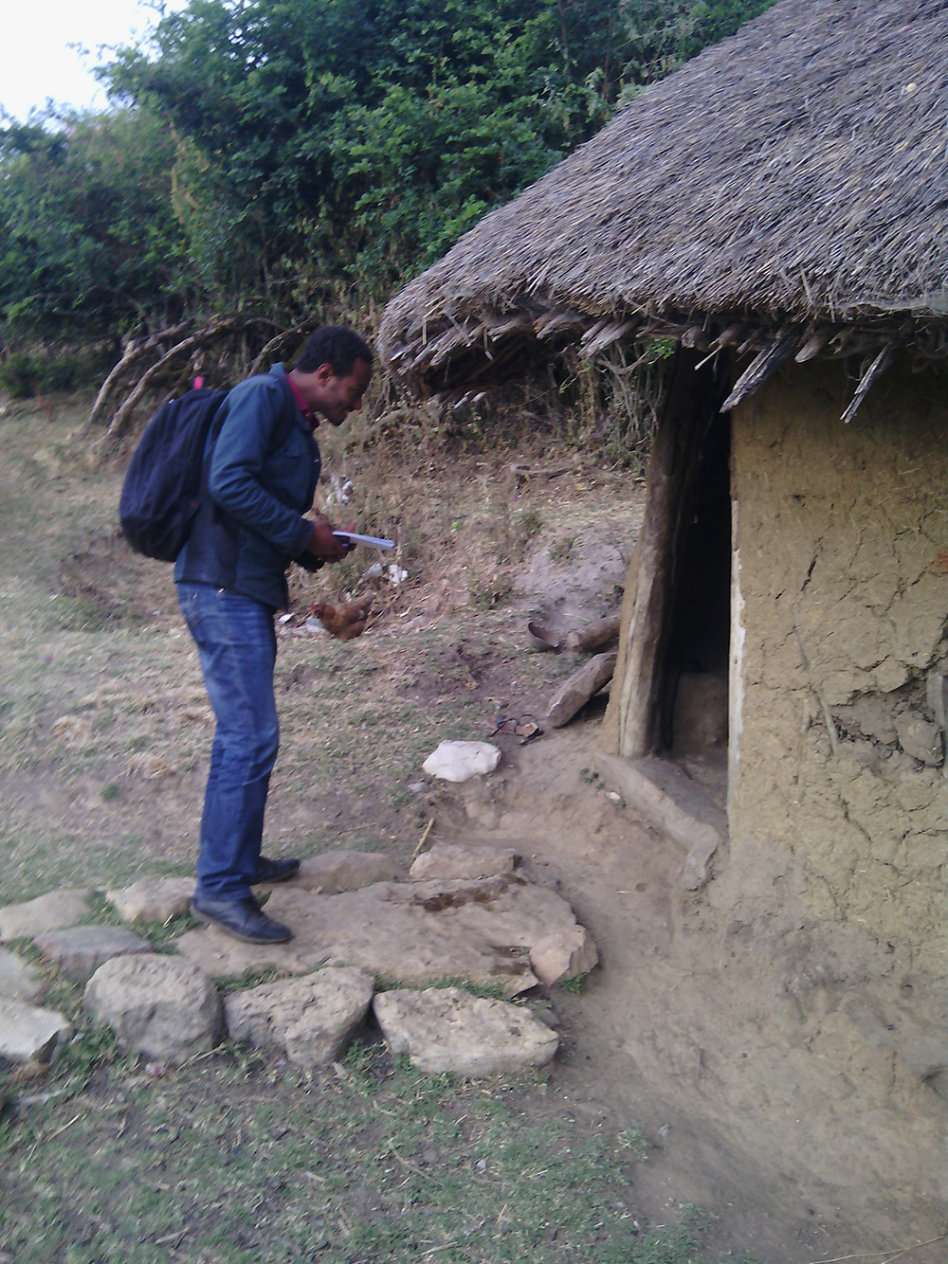
An interviewer arrives at a study participant's home to conduct household trials on antenatal calcium supplementation. © 2015 Zewdie Birhanu/Jimma University.

In each of 2 districts, we purposively selected 5 health posts for diversity in proximity to a referral health center and location in rural and semi-urban communities. We included healthy pregnant women aged 16 or older who were between 12 and 28 weeks’ gestation at enrollment and who would be available for interviews scheduled over the 6-week study period. Exclusion criteria were high-risk pregnancy, high habitual calcium intake, intellectual disability, and severe illness.

Health extension workers at each health post were informed of the study purpose and asked to identify all pregnant women in the catchment area who had and had not attended ANC. Based on health registries and their knowledge of pregnant women in the community, the workers provided information on demographic characteristics to facilitate purposeful selection of women representing variation in ANC use, gravidity, education, and daily hours spent outside the home. Purpose-ful sampling is widely used in qualitative research to select information-rich cases and identify samples that include key characteristics expected to influence experiences and perspectives of respondents.^42^

Selected women were screened using a questionnaire that included a brief 1-week food frequency measure focused on high-calcium foods. A nurse accompanying the study team followed standard antenatal history protocols to interview women and estimate gestational age based on last menstrual period. If eligible, women were invited to participate in the study, with invitations continuing until 5 women were enrolled in each of 10 communities. Five women were excluded after screening: 3 had an intellectual disability or serious illness, 1 was in the first trimester of pregnancy, and 1 planned to travel and would not be available to complete the study. No women were excluded for high calcium consumption or high-risk pregnancy.

For the quantitative comparison of adherence to calcium supplementation across the 3 regimens (Table 1), we estimated that a sample size of 15 women per group (N=45) would allow detection as significant a difference in intake between groups equal to 1 standard deviation (i.e., effect size = 1) with a 2-sided test, 80% power, and alpha of .05. We recruited 50 women to allow for 10% loss to follow-up.

### Intervention Materials

Based on formative research,^21^ we knew that women often felt they received inadequate information about why and how to take antenatal supplements. We adapted counseling cards from IFA materials designed by Nutritional International-Ethiopia and developed a reminder calendar for women to use at home, with motivating messages and illustrations indicating how many supplements to take and when to take them.

To allow for personal preferences, we offered a choice of 2 calcium carbonate products (conventional and chewable). The conventional (hard) calcium supplement was Ostocal D (Eskayef Bangladesh Ltd.), which contained 500 mg of elemental calcium and 200 IU of cholecalciferol. The chewable calcium supplement was Ideos (Innothera Chouzy Ltd.), which contained 500 mg of elemental calcium and 400 IU of cholecalciferol. Women who did not have IFA from ANC visits to health posts were provided supplements (Medicamen Biotech Ltd.) containing 60 mg of elemental iron and 400 μg of folic acid.

### Data Collection Procedures

Five trained interviewers recruited 50 women, who were approached at home and screened for eligibility. Participants were randomly assigned to 1 of 3 supplementation regimens (Table 1). Allo-cation was distributed evenly across community and interviewer.

Participants were offered daily calcium and IFA supplementation for 6 weeks and interviewed on their experiences every 2 weeks (range: 13–15 days), totaling 4 interviews (1–1.5 hours each) per participant. Interviews were conducted using semistructured interview guides that had been translated into Afan Oromo, back-translated, and pretested in the local context.

Table 2 summarizes the sequence of activities. During visit 1, interviewers assessed a woman’s ANC and IFA supplementation experience and counseled her on the benefits of supplements, regimens, side effects, and adherence strategies using counseling cards and reminder calendars. Conventional and chewable supplements were presented so that women could examine them and choose the preferred supplement type, discussing the reasons for their choice. Interviewers asked about women’s willingness to try supplementation for 2 weeks until the next interview and addressed any concerns. Counseling was the same for all regimens, except for the regimen-specific details on how often to take supplements. Women were also asked about their views on asking a family member or friend to remind and encourage their supplement use; results on acceptability of adherence partners are reported elsewhere.^43^

**TABLE 2.** Sequence of Activities for Trials of Improved Practices Testing Acceptability of Antenatal Calcium and Iron-Folic Acid Supplementation in 2 Rural Districts, Ethiopia

		**Visit 1**	**Visit 2**	**Visit 3**	**Visit 4**
		**(Enrollment)**	**(2-wk follow-up)**	**(4-wk follow-up)**	**(6-wk follow-up)**
Intervention	Information provided	Counseling, reminder materials	Supportive	Supportive	Supportive
	Regimen	Random assignment	Maintained	Choice	Choice
Data collection	Acceptability response	Willing to try?	Tried? Willing to continue?	Continued? Willing to continue?	Continued? Willing to continue?
	Qualitative	Anticipated challenges/facilitators Motivations Concerns	Experiences Challenges/ facilitators Motivations Concerns Others’ reactions Strategies	Experiences Challenges/ facilitators Motivations Concerns Others’ reactions Strategies	Experiences Challenges/ facilitators Motivations Concerns Others’ reactions Strategies
	Quantitative	Demographics	Adherence (MEMS)	Adherence (MEMS)	Adherence (MEMS)

Abbreviation: MEMS, medication event monitoring system.

Follow-up visits 2, 3, and 4 involved interviews about supplementation experiences, concerns, challenges, and facilitators. At the end of each interview, women were asked if they were willing to continue with supplementation; their interview responses were tabulated to assess acceptability.^44^ At each visit, participants also had the option to choose either conventional or chewable calcium supplements for the next period and were provided with supplements; this allowed us to assess preference for calcium products.

**Figure:**
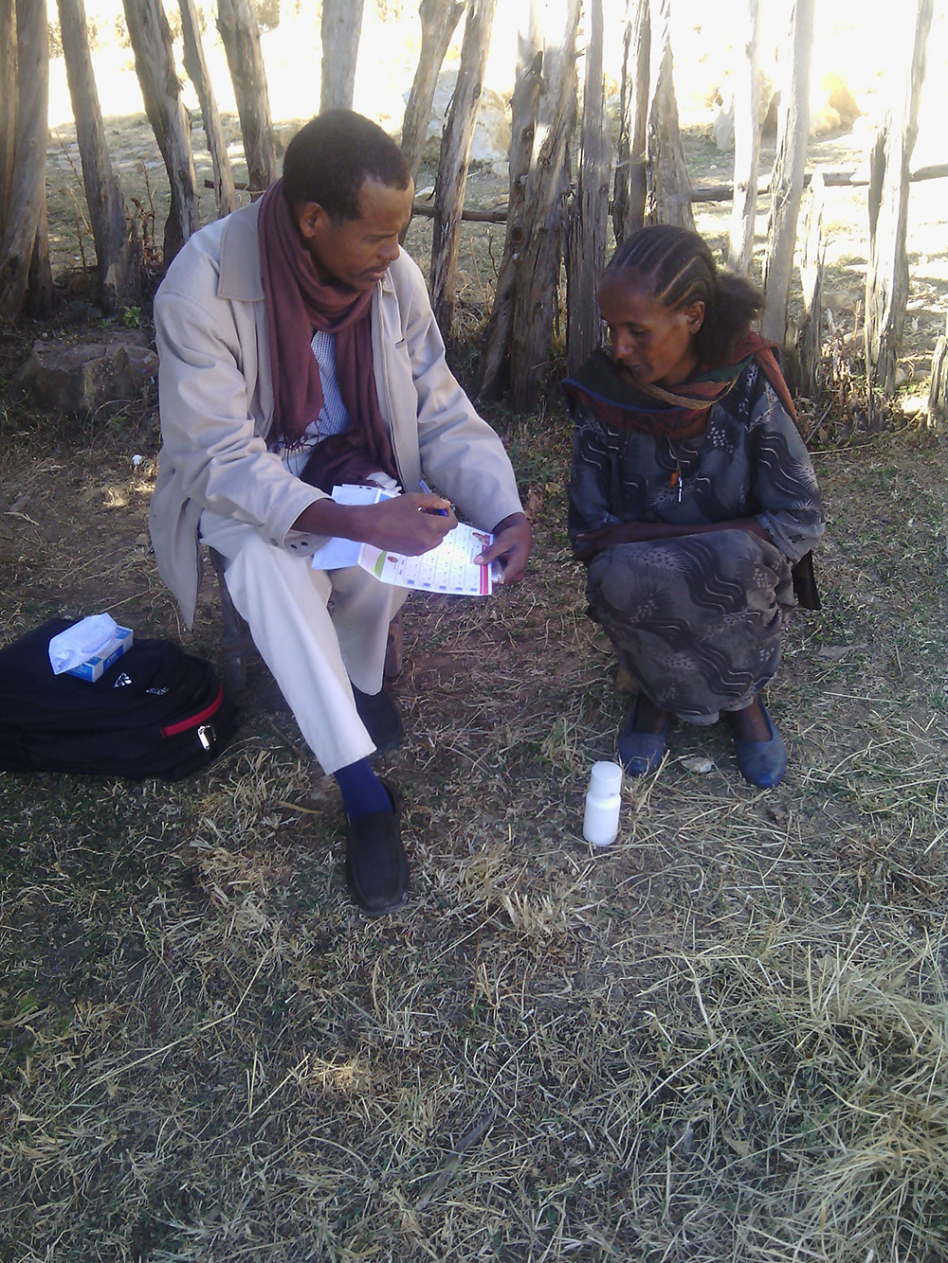
A trained interviewer conducting an interview with a pregnant woman about calcium supplementation acceptability and adherence. © 2015 Zewdie Birhanu/Jimma University.

At visit 3, after 4 weeks on a randomly as-signed regimen, women were also offered the option of choosing whichever regimen they preferred to follow, and then asked to discuss their reasons. This discussion provided deeper insight into different women’s responses to the 3 regimens, and the final interviews on visit 4 focused on the women’s experiences with the regimens they chose. On visit 4, women were also asked if they were willing to continue supplementation after the study ended, and if so, they were given supplements to last the remainder of their pregnancy.

Adherence to calcium over the previous 2 weeks was evaluated at visits 2, 3, and 4 using medication event monitoring system (MEMS; AARDEX Ltd., Zug, Switzerland) technology with a microelectronic chip registering the date and time of each bottle opening. Bottle openings were the proxy for intake of 1 supplement. Participants were instructed to (1) only open the bottle to take 1 supplement; (2) keep the bottle away from children or others who may open the bottle; and (3) take the bottle when traveling. IFA (and calcium) adherence was additionally captured via self-report. At each visit, women received 40–60 calcium supplements (i.e., an amount slightly exceeding regimen requirements), and those who did not have IFA from ANC also received 2 10-capsule IFA blister packs.

Participants received calcium and IFA supplements and counseling on benefits, side effects, and adherence strategies.

### Data Analysis

Interviews were audio-recorded, transcribed verbatim, and translated into English by each interviewer. ZB reviewed each interviewer’s first audio recording during each interview round against the transcript for accuracy of translation and quality of interview technique, and held daily debriefs to discuss challenges to the protocol. ZB and YK reviewed all transcripts for clarity and completeness.

GCK, SEO, and SLM independently read and coded 16 transcripts using a grounded theory approach to develop initial code categories based on participant experiences with supplementation.^45^ Investigators jointly reviewed codes and developed a codebook^46^ to guide systematic coding of transcripts in Atlas.ti version 7 (Scientific Software Development GmbH). GCK and SEO independently coded 30% of transcripts to standardize coding. All authors discussed and resolved differences in coding and code definitions. Results were organized into matrices summarizing participant experiences, preferences, and key quotations to allow comparison across cases and within cases.^47^ Matrices and relevant output were shared with the research team to review consistency of coding and reach consensus on interpretation. Qualitative results are summarized in the narrative, describing approximate proportions of respondents who mentioned a point to give a sense of salience. Lists of reasons begin with the most commonly mentioned. Similar topics were discussed across all time points, so interview results from all visits are summarized by theme, noting changes in experiences over time. Illustrative quotes are included in the text and in a table on barriers and facilitators.

MEMS data were configured using Powerview (MWV) and cleaned using SAS/STAT, version 9.3 (SAS Institute Inc.). Percent adherence (or “adherence rate”) was defined as the number of MEMS events divided by number of prescribed doses during the assessment period multiplied by 100, and capped at 100% (capping occurred for 11% of MEMS measurements). Daily consumption is reported in milligrams and was defined as the number of MEMS events multiplied by 500 mg, divided by days in assessment period. The Assessment period was defined as the number of days between interviews. MEMS events record-ed on interview days were not included in the analysis to exclude events resulting from demonstration and practice of bottle opening. One-way analysis of variance (ANOVA) was used to compare adherence rates and calcium consumed (mg) across 3 regimens. Significance was set at *P*<.05. The post-hoc Tukey HSD test was used to determine which group means were significantly different from each other. Analyses were performed using SPSS 24.0 (IBM Corp.). MEMS data were triangulated with self-reported adherence to categorize women by adherence level and inform interpretation of interview data.^48^^,^^49^

### Ethical Approval

Cornell University’s Institutional Review Board (1205003071), the Ethiopian Public Health Institute, and the Oromia Regional Health Bureau approved this study. All study staff completed training on research ethics, protocol, and informed consent. All women approached agreed to participate and gave written informed consent.

## RESULTS

Forty-nine of 50 recruited women (98%) completed the 6-week study; 1 participant withdrew after 2 weeks for unknown reasons. Another woman completed the study but had incomplete data so was dropped from analysis. Results are reported for the final sample of 48 women with complete data.

### Participant Demographics

All but 3 participants were married and mean gestational age was 20.5 weeks (range 13–28 weeks). The majority were rural; most households owned livestock (73%) and grew crops (71%). Most women routinely spent time away from home (77%); a third spent over 5 hours away daily. Women’s educational levels were very low, and food insecurity was relatively common (Table 3). Demographic characteristics were similar across groups assigned the 3 regimens, although the 2-event group tended to be more food secure (Table 3).

**TABLE 3.** Characteristics of Pregnant Women Who Completed Household Trials on Calcium and Iron-Folic Acid Supplementation in 2 Rural Districts, Ethiopia (N=48)

	**4-Event** **Regimen** **n=15**	**3-Event** **Regimen** **n=17**	**2-Event** **Regimen** **n=16**	**Total** **Sample** **N=48**
Maternal age, y, mean (SD)	28.5 (4.5)	26.0 (6.6)	26.4 (6.0)	26.9 (5.8)
Gravidity, No. (%)				
Primigravida	4 (26.7)	5 (29.4)	4 (25.0)	13 (27.1)
Multigravida	11 (73.3)	12 (70.6)	12 (75.0)	35 (72.9)
Educational level, No. (%)				
None	8 (53.3)	7 (41.2)	6 (37.5)	21 (43.8)
Some primary	5 (33.3)	7 (41.2)	8 (50)	20 (41.7)
Completed primary or some secondary	1 (6.7)	1 (5.9)	1 (6.3)	3 (6.2)
Completed secondary or higher	1 (6.7)	2 (11.8)	1 (6.3)	4 (8.3)
Household food security,^a^ No. (%)				
Secure	5 (33.3)	5 (29.4)	9 (56.3)	19 (39.6)
Mildly insecure	2 (13.3)	2 (11.8)	3 (18.8)	7 (14.6)
Moderately insecure	5 (33.3)	5 (29.4)	3 (18.8)	13 (27.1)
Severely insecure	3 (20)	5 (29.4)	1 (6.3)	9 (18.8)

a FANTA: Household Food Insecurity Access Scale (HFIAS) for Measurement of Food Access: Indicator Guide (https://www.fantaproject.org/monitoring-and-evaluation/household-food-insecurity-access-scale-hfias).

### Antenatal Care and IFA Usage Before Study

At visit 1, about 70% of respondents had attended ANC once or twice during the current pregnancy, on average starting at 3.5 (range: 1–6) months; a few had attended more often and almost 20% had not attended. Three-fourths of respondents received IFA before study enrollment, with most taking IFA for <1 month. Several women mentioned side effects and methods to minimize them; only 3 had discontinued use. Despite programming in the study sites to increase access to IFA information, women wanted more specific details:

*I want to know the detailed importance for mother and fetus. The health worker said, “Take 1 pill daily and come back on your appointment” but this is not enough.* —28-year-old woman, 4-event, very high adherence

### Willingness to Try Calcium and IFA

Across visits, all 48 women were willing to try or to continue calcium and IFA supplementation. At visit 1, when asked about potential difficulties with supplementation, most women said they did not expect barriers, anticipating the health benefits would motivate them to consume supplements as counseled. Six women, despite their willingness to try supplementation, initially did not adhere. Their adherence improved following clarification on visit 2 to ensure they understood the schedule and that they could take a missed supplement when they remembered instead of skipping a dose.

Initially, more than one-fourth of the women anticipated 1 or more barriers, including forgetting during work or travel, having side effects or feeling ill, taking too many supplements, or not having water or food available. A few women preferred to keep calcium in a discrete place for fear others would assume it was antiretroviral therapy.

When asked what might facilitate adherence, nearly half of the women suggested keeping supplements in a visible place, about one-fourth suggested taking calcium with meals, and 9 women suggested reminders from family members.

Counseling women on the need to take calcium was challenging because women were not aware of preeclampsia or hypertensive disorders of pregnancy. Many women knew about hypertension in general, describing it as “increased amount of blood,” but only 1 participant associated it with pregnancy. Most women knew of anemia, half referring to it as “low blood” and IFA as the tablet that “fills blood.” A few women initially expressed uncertainty about taking calcium and IFA together for what seemed to them to be opposing health conditions, and further counseling was required for these women. Women who understood supplementation as a preventive measure accepted the need to take both calcium and IFA.

Counseling women on the need to take calcium was challenging because women were not aware of preeclampsia or hypertensive disorders of pregnancy.

### Barriers and Strategies for Improving Adherence

Qualitative analysis of reported adherence barriers and facilitators did not identify notable differences across women with high and low adherence, although there were some differences by assigned regimen. Quotes illustrating the barriers and facilitators discussed below are included in Table 4.

**TABLE 4.** Quotations Illustrating the Range of Perceived Facilitators and Challenges for Adherence to Calcium Supplementation Across High and Low Adherers, Ethiopia (N=48)^a^

**Facilitators**	**Illustrative Quotations**
Reminders	*I used the calendar … I post it on the wall and always when I see it, I remember to take my pills.* —25-year-old woman, 4-event, very high adherence *My husband goes to work during my pill-taking times, which helps me remember. I also associate taking pills with mealtimes. —*22-year-old woman, 3-event, increased adherence
Value of prevention	*I often think, “If I die from some problem, what would happen to my children?” I took these pills as you told me to prevent such risks. —*27-year-old woman, 3-event, high adherence *Other people may say, “Why do I bother to take the pills if I am healthy?” Those who are sick are more concerned with taking pills, but I keep taking them to help my baby survive. —*38-year-old woman, 3-event, very high adherence
No adverse effects and relief of symptoms	*Feeling healthy and comfortable helped me take [calcium]. If I were not happy in taking the pill, I couldn’t take it. I may avoid it even. —*37-year-old woman, 3-event, very high adherence *Before I started to take these pills I had stomach aches and back pain. Now I am free from these problems. I like these pills very much due to the benefits I received. —*30-year-old woman, 2-event, high adherence
Self-efficacy	*My brothers asked me whether [calcium] is for [HIV/AIDS]. I don’t want to fear what other people think. I will use it without fear; it is about my health, not others.* —30-year-old woman, 3-event, high adherence
Travel strategies	*I took out one [calcium] pill and covered it with clean paper to bring with me. To take one pill, I do not want to carry all the pills in the bottle. If I carry the bottle, people may see it … attracting their attention. —*22-year-old woman, 4-event, very high adherence
Trust in government and health services	*I am taking the pills consistently. People talk about the pills fattening the fetus… or other unjustified ideas … but health workers and government give this service for our benefit. —*20-year-old woman, 2-event, very high adherence *Since the government does not give us anything which hurts us, taking these pills is not difficult.* —30-year-old woman, 3-event, adherence decreases
Counseling and follow-up support	*Your advice has also greatly influenced me to take the pills because I am convinced about their importance and I was committed to take the pills myself. —*20-year-old woman, 3-event, high adherence *Had I not received your advice on how to take [calcium] I could have stopped taking it. And I would have thrown away [IFA] because of the severe heartburn. After your advice to take it right before sleeping, there is no problem for me. —*30-year-old woman, 4-event, very high adherence
**Challenges**	**Illustrative Quotations**
Forgetting	*It was difficult to take [calcium] every day because we are busy with harvest these weeks and I forget to take them.* —18-year-old woman, 3-event, high adherence *Taking [calcium] in the morning is difficult—breakfast may even be delayed—because when I wake up I directly deal with work and may forget it. —*38-year-old woman, 2-event, decreased adherence
Away from home during the day	*Taking [calcium] midday is difficult. I went to visit my parents. I left in the morning but failed to come back the same day. I did not take pills for two days. To overcome this, I have to take the pills with me.* —24-year-old woman, 4-event, very low adherence *Midday, most times I am not at home. I may visit family whose relative died, attend a wedding, or travel elsewhere. In the morning no one leaves without breakfast and in the evening it is a must to come home for dinner, but it is unsuitable to take pills at lunchtime.* —26-year-old woman, 3-event, very high adherence
Stigma and community perceptions	*People do not know [calcium]. They say, “During our pregnancy we took [IFA] but we don’t know this one.” People may assume this pill is given for [HIV/AIDs]. This pill is difficult for me because I don’t think people’s presumption will change and I don’t know what to do.* —25-year-old woman, 4-event, very low adherence *Taking these pills is very difficult. People who have seen me taking pills for a long time may suspect I am taking [HIV/AIDs] drugs. I can convince he who dares to ask me, but he who doesn’t know me and doesn’t ask, I don’t have the chance to explain the reality.* —37-year-old woman, 4-event, very high adherence
Side effects	*I didn’t take [calcium] for the last week. I frequently felt nauseous and abdominal discomfort so I stopped. I feel real discomfort in taking it. I don’t feel healthy. —*25-year-old woman, 4-event, very low adherence *When I take [calcium] I tend to vomit. It nauseates me. I drink a lot of water and then swallow it. Without water it would have all come back up. I really took this pill with great difficulty … I took only for the sake of its benefit. —* 20-year-old woman, 3-event, high adherence
Lack of food	*Most of the time I can’t find food in the morning. If I find a little food, I have to give priority to my kids. I do not want to take [calcium] if I am unable to find at least one mouthful. You told me to take it if I am unable to find food, but I’m afraid of taking pills like that. —*33-year-old woman, 4-event, decreased adherence *I haven’t stopped taking the pills. Even though I couldn’t get adequate and quality food, I took it with whatever food was available. —*24-year-old woman, 2-event, very high adherence
Illness	*I was sick. I couldn’t eat and I even drank coffee with difficulty. Now I have my health back. I can eat and do my work well. This is why I remember to take the pills. —*27-year-old woman, 4-event, increased adherence
Large baby	*People in our community say during pregnancy pills fatten the fetus. This was discouraging me, but I think its advantage outweighs the disadvantage and I decided to take the pills.* —30-year-old woman, 2-event, very low adherence *My worry is the pills may fatten the fetus and cause problems during delivery. People in our community said this… I think it is [calcium], because it has milk content, but people say [IFA] can also fatten the fetus… I was taking it expecting its benefit outweighs this problem. I was waiting for you to clarify the reality.* —28-year-old woman, 4-event, very high adherence

Abbreviation: IFA, iron–folic acid.

a Participants categorized by mean adherence rate to assigned regimen: over one-third (20/48) were “very high” adherers (90%–100%), 9 were “high” adherers (75%–89%), 4 were “low” adherers (45%–74%), and 3 were “very low” adherers (<45%). Twelve women did not fit these categories due to substantial changes in adherence over time: for 7 women adherence was categorized as “increased” and for 5 women adherence “decreased.”

Forgetting supplements when busy with work, chores, or children, or when away from home was the most common barrier to adherence, reported by one-third of the women. The midday tablet in the 4- and 3-event regimens was the most difficult to take consistently, particularly on days with extra activities (e.g., church, market, holidays, weddings) and for women who were responsible for livestock or harvesting. Forgetting decreased over time, and women reported that habituation to the regimen helped them to adhere. As the study progressed, the harvest season ended and more women were at home, which helped adherence, particularly the midday dose.

Most women who reported missing doses when outside the home had thought they would return in time; they suggested they could solve this problem by carrying the bottle, although most did not report trying this. One-fourth of the women mentioned not taking calcium supplements in public and concealing calcium to avoid questions about the supplements or because they had been discouraged from taking calcium by others who viewed it as HIV/AIDS medication. Five women reported that this possibility affected their adherence; several others did not care what others thought. Women acknowledged that lack of community awareness and experience with calcium caused misperceptions. A few women said that taking supplements in public was inappropriate, particularly during church, burial ceremonies, or other formal events, or during fasting periods, causing them to occasionally miss doses. Women were counseled to take only 1 calcium tablet when opening the bottle so MEMS could successfully record consumption. However, women who took calcium out of the bottle to carry for later usage were less likely to miss midday doses.

Women acknowledged that lack of community awareness and experience with calcium caused misperceptions.

A third of participants experienced side effects that they attributed to calcium (nausea, light-headedness, vomiting, abdominal distension, and constipation) and almost half experienced side effects they attributed to IFA (heartburn, nausea, gastritis, abdominal cramp/distension, headache, and weakness/dizziness). Seven women skipped calcium doses due to side effects; 5 discontinued use. Nine women missed IFA doses; 6 discontinued use. Skipping doses due to calcium side effects declined for 60% of these women by visit 4.

Several women mitigated side effects or unpleasant taste or smell by consuming more water and food with supplements, and some took IFA right before bed or ate something sweet, such as sugarcane, afterwards to reduce the bitter taste and nausea. Two women said that taking calcium concurrently with IFA mitigated IFA side effects. Four women reported that side effects went away on their own. Another 9 women continued supplementation with some discomfort, persevering to obtain the health benefits. Some women noted they took calcium with ease and no adverse effects.

Counseling materials recommended taking supplements with food and water to minimize side effects, but lack of food and water at dosing times made this difficult for more than 12 women, affecting adherence to calcium or IFA. This difficulty was especially true for women reporting long work hours outside the home or food insecurity. Women reported reserving a small amount of food (e.g., 1 piece of bread) to consume with supplements. One woman requested small amounts of food from her neighbors to take the supplements during a food shortage.

Six women reported skipping calcium and IFA supplements due to illness (kidney problems, gastritis, cough, and weakness); 3 were prescribed medications by a health worker and felt it was too difficult to continue supplementation while taking medication. Adherence usually improved when women regained their health.

When asked if anyone discouraged supplement adherence, many women said family members or others linked supplements with large fetuses and difficult deliveries. Most women felt this discouragement was “unjustified” or “lacked evidence” but 6 were concerned it might be true.

### Facilitators to Adherence

When asked what helped women remember their tablets, three-fourths of the respondents said reminders from family members and the reminder calendar, including encouragement from others to use the calendar or husbands or children marking the calendar when supplements were taken. A few women used the calendar to explain supplementation to others. Keeping pills visible and taking supplements during routine events (e.g., mealtimes or coffee breaks) also helped many women adhere.

Despite an array of adherence barriers, especially with the midday calcium dose, calcium and IFA supplementation proved highly acceptable.

Most women were motivated by perceived health benefits from calcium or IFA supplements, especially relief from light-headedness, dizziness, and headache, as well as increased strength, appetite, and fetal movement. Over time, more women reported that anticipated and experienced health benefits facilitated their adherence. Over a third of the women said they were motivated to take calcium and IFA to supplement what they viewed as their own poor diets, restating counseling messages used by interviewers that tablets provided nutrients that otherwise needed to be obtained from foods they could not regularly access (e.g., milk, liver, or meat).

Over half of the women mentioned trusting that health workers and the government would only provide tablets that were good for them. This trust did not correlate with adherence, due to the influence of other barriers (e.g., forgetting, side effects), but strongly motivated overall acceptance of supplementation in this context. Similarly, many women noted that the counseling and advice received from health workers and the study interviewers encouraged them to continue taking the supplements, especially when they had doubts or challenges.

Counseling, family support, self-efficacy, and home-based reminders helped women adhere.

### Calcium Product Preference and Acceptability

At study enrollment, about three-fourths of the women selected chewable supplements for their sweet taste and smell, and sometimes for ease of consuming without water and a belief that conventional tablets are difficult to swallow. Women who initially chose conventional supplements cited simplicity of use, absence of strong taste, smaller size, and concern that chewable supplements could cause nausea.

Over half of the women maintained their original choice throughout the study, citing satisfaction with supplement properties, absence of challenges or side effects, familiarity, and dislike or uncertainty about the other supplement type. However, about a third of the women who selected chewable supplements switched to conventional due to untoward effects (e.g., excessively sweet taste and foamy mouth) or side effects (nausea, vomiting, and heartburn). Four women who changed from conventional to chewable supplements cited lack of access to water; 1 woman disliked the taste. Most who switched supplement type reported full correction from previous challenges. At final interviews, among the participants willing to continue calcium until delivery, about 60% chose chewable supplements.

### Regimen Preference

Although regimens were originally randomly assigned, at visit 3 participants were shown illustrations of 3 dosing regimens and asked to choose a regimen for the final 2 weeks. The 2-event was preferred; nearly all participants assigned this regimen (88%; 14/16) chose to continue at visit 3 compared with fewer than half (41%; 7/17) of the women assigned to the 3-event and very few (27%; 4/15) assigned to the 4-event. Overall, two-thirds of the women (20/32) switched to the 2-event versus 2 women switching to the 3-event and 1 switching to the 4-event. Women chose the regimen with the lower calcium dosage due to difficulties adhering to the midday supplement.

### Calcium Adherence and Consumption

MEMS data indicated that most women (42/48) initiated calcium supplementation immediately, and all women eventually tried supplementation. Across the sample, mean adherence rates after 4 weeks were high (approximately 80% for all regimen groups), with no associations between adherence rate and assigned or chosen regimen (Table 5). Throughout the study, most women (35/48) maintained average adherence rates of over 80% or increased their adherence to this level; a few (7/48) completed the study with a mean adherence rate of less than 50%.

**TABLE 5.** One-Way ANOVA for Antenatal Calcium Supplementation Adherence Rate and Daily Calcium Consumption, by Randomly Assigned Regimen (for 4 Weeks) and Chosen Regimen (for 2 Weeks) Among Ethiopian Women in 2 Rural Districts (N=48)^a^

	**4-Event**	**3-Event**	**2-Event**	***F*_2,45_**	***P* Value** ^b^
Assigned regimen (weeks 0–4)	n=15	n=17	n=16		
Adherence rate, mean % (SD)	77.1^a^ (34.3)	83.4^a^ (14.1)	81.1^a^ (22.7)	.265	.768
Calcium consumption, mg/d, mean (SD)	1,156.4^a^ (514.5)	1,251.1^a^ (211.9)	811.3^b^ (227)	7.53	.002
Chosen regimen (weeks 4–6)	n=5	n=9	n=34		
Adherence rate, mean % (SD)	95.9^a^ (3.9)	80.1^a^ (23.1)	73.8^a^ (32.8)	1.248	.297
Calcium consumption, mg/d, mean (SD)	1,438.5^a^ (58.3)	1,200.9^a^ (347.1)	738^b^ (328.3)	15.66	<.001

Abbreviation: ANOVA, analysis of variance.

a Groups followed by the same letter are not significantly different in post hoc multiple comparisons with Tukey correction, *P*<.05.

b *P* value for overall ANOVA *F*-test.

Women on the 2-event calcium regimen (1 g/d) did not achieve higher adherence rates and therefore consumed significantly less calcium than women taking 3 daily calcium doses.

A one-way ANOVA showed a significant difference in the mean calcium consumption between randomly assigned regimen groups, but not in adherence rate. Post hoc comparisons using the Tukey HSD test (Table 5) indicated that women assigned the 2-event regimen on average consumed significantly less calcium (811.3±227 mg/d) than women assigned the 3-event (1,251.1±211.9 mg/d) or 4-event (1,156.4±514.5 mg/d). Women assigned the 3-event regimen consumed the most calcium because of the 1,500-mg dose combined with the highest adherence rate, but the trend was not statistically significant. In the later phase in which women chose their regimen, results were similar; calcium consumption was lower among women who chose the 2-event regimen and adherence rates did not differ. Statistical results for this later phase exploring women’s choices must be interpreted with caution given the wide range in group sizes due to the self-selection that was inherent in giving women a choice.

Although the 2-event regimen was preferred, switching to this regimen did not improve the adherence rate, so mean daily calcium consumption for the 20 women making this selection decreased from 1,134.6 mg (±454.4) to 752.8 mg (±327.8). Table 6 shows the trends in mean daily calcium consumption between women’s assigned and chosen regimens. Adherence was generally high, thus most women consumed on average at least 800 mg/d regardless of regimen type. Poor adherers tended to choose the 2-event regimen yet continued to struggle with adherence, yielding < 500 mg daily consumption for 25% of this subgroup. While some women reported that aspects of regimens were difficult to adhere to, having a choice of regimen did not substantially affect adherence. All women who were assigned or chose the 3-event regimen consumed on average at least 500 mg/d; most consumed at least 800 mg. Women selecting 3- and 4-event regimens were already highly adherent with no notable side effects and believed the new regimen with additional calcium would increase health benefits.

**TABLE 6.** Trends in Average Daily Calcium Intake at 4 Weeks (Assigned Regimen) and 6 Weeks (Chosen Regimen) Within Household Trials Among Participating Ethiopian Women in 2 Rural Districts (N=48)^a^

	**Assigned regimen** **(weeks 0-4)**			**Chosen regimen** **(weeks 4-6)**		
Calcium consumed, mg/d^c^	4-event (n=15),^b^ %	3-event (n=17), %	2-event (n=16), %	4-event (n=5), %	3-event (n=9),^b^ %	2-event (n=34), %
<500	13	0	6	0	0	26
500 to <800	7	12	25	0	11	3
800 to <1,000	7	0	50	0	11	56
1,000 to <1,500	47	82	19	60	44	15
1,500	27	6	0	40	33	0

a Cut-points based on 500 mg calcium per supplement.

b Because of rounding, percentages may not total 100.

c Estimated average requirement (800 mg/day) and recommended dietary allowance (1000 mg/day) for pregnant women aged 19–50 years based on *Dietary Reference Intakes for Calcium and Vitamin D.*^50^

At study end, almost all women were willing to take calcium and IFA until delivery and three-fourths chose the 2-event regimen. Five women were unwilling to continue calcium and 4 were unwilling to continue IFA. Among women discontinuing calcium, 3 had low mean adherence throughout the study (12%–46%); 1 woman was 100% adherent but had severe heartburn and nausea; and 1 woman (87% adherent) felt she had taken enough.

## DISCUSSION

Hypertensive disorders in pregnancy are a leading health problem in Ethiopia, and early screening and prevention strategies are needed.^51^^,^^52^ In response to WHO guidelines,^7^ our study explored modifiable factors associated with adherence to calcium supplementation and identified strategies to optimize implementation of calcium with IFA supplementation.

Women in Oromia, Ethiopia, found antenatal calcium and IFA supplementation acceptable. All participants were willing to try supplementation and most adhered to calcium supplements at relatively high rates over a 6-week period, after receiving supplements and behavior change communication to motivate and support adherence. Perceptions of physical benefits and the need to supplement poor diets were motivating, and social support and reminder materials helped to overcome barriers and enhance adherence.

Provision of appropriate counseling and home-based reminders can potentially motivate adequate adherence to antenatal calcium supplements integrated into IFA supplementation regimens.

A comparison of our results with parallel research in Kenya^53^ identified important similarities and differences across settings. Calcium consumption and adherence rates in Kenya were generally lower. Most participants in Ethiopia and Kenya (∼75%) preferred chewable supplements; however, in Ethiopia, the difference in preference at the end of the trial was negligible. Results in Ethiopia indicate that a sweet chewable tablet did not strongly influence adherence and, given the significantly higher cost, inclusion of this option in programs is not warranted. However, preferences are likely to vary by context.^23^^,^^53^

Side effects of calcium or IFA were widely reported, yet only affected adherence for a subgroup of the women, in contrast to the reported impact of side effects (or fear of them) on IFA adherence in previous Ethiopian studies.^54^^,^^55^ Counseling and regular follow-up visits as part of the TIPs approach may have positively influenced women’s adherence, especially given high levels of trust in medical providers in Ethiopia.^17^ Women who know the importance of IFA are more likely to adhere^21^^,^^54^^,^^56^; however, our results and previous formative research indicate that follow-up counseling to monitor adherence and address challenges such as side effects is as important as initial education.^21^ Utilizing extension officers as agents for sharing information on antenatal supplements has been recommended,^21^ and continued support of the program could leverage their home visits and trusted relationships to follow-up on supplementation.^57^ As in formative research,^21^ counseling on supplements for prevention and as a dietary supplement led to increased motivation and clarified need for concurrent use of IFA for “low blood” and calcium for “high blood.”

In light of expected low adherence to complex supplementation regimens requiring women to take pills 3 to 4 times a day, we anticipated that a reduced supplementation schedule would be easier to adhere to, such that calcium consumption might be equivalent. Although most women preferred taking 2 instead of 3 daily calcium supplements, choice of dosing regimen did not significantly affect adherence. In neither Ethiopia nor Kenya did 2-event regimens (1 g/d) increase adherence rate enough to result in calcium consumption comparable to regimens of 1.5 g. Research is lacking on the minimum effective dose for preventing preeclampsia. Some evidence suggests low-dose calcium supplementation (<1 g/d) can reduce pregnancy-induced hypertension risk,^8^^,^^36^^,^^58^ even with supplementation of ≤600 mg/d, which is lower than the mean calcium consumption for the 2-event regimen in this study. Thus, while women understandably prefer a 2-event regimen, additional randomized controlled trials (such as ongoing trials in India and Tanzania)^59^ are needed to assess whether it results in consumption levels adequate to reduce preeclampsia risk. Women’s choices might change if counseled that the 3-event regimen would be significantly more effective.

If a 3-event regimen (1.5 g/d) is necessary to reduce preeclampsia risk, it is important to consider how to overcome barriers to calcium supplement adherence that were the most limiting for the midday dose when women were engaged in activities or outside the home. Health workers need to be aware that taking supplements 3 to 4 times daily is difficult, and discussing adherence strategies (especially for remembering when outside the home) is as important as discussing regimen and side effects during counseling. Women reported using the reminder calendar as a visual cue, and frequent follow-up visits helped them adhere. Other counseling messages women found useful included placing supplements in a visible location, taking supplements during routine events (e.g., mealtimes), and asking for support from relatives, as found previously.^17^^,^^53^ In this study, familial support, such as provision of simple reminders, encouragement, and bringing supplements and water, was the largest facilitator of adherence and is described in detail elsewhere.^43^ Other studies recommend active involvement of family members to enhance adherence to micronutrient supplements and improve maternal diet.^26^^,^^43^^,^^60^

The Ethiopian government has highlighted prenatal calcium supplementation in national health policy, but it has yet to be implemented as a part of ANC.^61^ Our research suggests the importance of targeting communities, not just pregnant women and their families, when expanding antenatal supplementation programs. Over 40% of the sample mentioned stigma due to pills being associated with HIV/AIDS or concern that supplements cause excessive fetal growth and difficult deliveries, beliefs noted previously in Ethiopia.^17^^,^^21^^,^^55^ Discouragement often came from individuals not typically targeted for antenatal supplementation counseling, including extended family members, neighbors, and other community members. Broad community-level sensitization on calcium and IFA supplementation could help normalize taking multiple tablets during pregnancy as a preventive measure, particularly where preeclampsia is not well known.^21^ Community-based mobilization to promote IFA supplementation has been successful elsewhere.^62^

Strategies for improving adherence were discussed during counseling and were more effective than reduced dosage for boosting adherence.

The level of calcium supplementation must be considered in relation to dietary calcium intake during pregnancy. In Ethiopia, dietary calcium intake during pregnancy is often low.^63^ However, dietary intakes are variable such that the preferred 2-event regimen could be sufficient for some women to meet requirements, and higher doses could exceed the tolerable upper intake level.^64^ Programs that strengthen dietary quality and diversity should also be considered and, if feasible, may be more sustainable. Consumption of teff, a common local grain,^65^ and diverse diets^66^^,^^67^ have been associated with anemia reduction in Ethiopia and dietary diversity lowered preeclampsia risk in other populations.^68^^,^^69^ Diversifying diet in addition to IFA supplementation was associated with reduced occurrence of symptoms suggestive of preeclampsia and eclampsia in India.^70^

### Strengths and Limitations

The TIPs approach gathers data on emic views and actual experience with antenatal supplements and can provide effective guidance to strengthen nutrition programs.^43^^,^^53^^,^^60^ However, home delivery of supplements, repeated counseling during household visits, and purposeful sampling limits generalizability of the findings. This intensive model facilitated in-depth exploration of the complex factors affecting acceptability and adherence, but facility-level implementation research is needed to determine feasibility and sustainability through the health system in Ethiopia and in other contexts. Researchers undertook a larger, facility-based study in Kenya in order to further explore findings from TIPs.^71^

Use of electronic monitoring in combination with interview data to assess participants’ adherence strengthened study conclusions. Agreement between MEMS and self-report data varied across the sample, so our report focused on MEMS adherence data, which appeared to be more accurate. For example, most women whose adherence (measured by MEMS) was categorized as low or very low or whose adherence decreased throughout the study regularly stated that they never or only rarely missed a supplement and that taking calcium was “easy.” MEMs allowed for cross-checking against this self-report data. Upon further probing, 8 women reported different (and multiple) barriers. Consistent overreporting of product use is well documented^72^^,^^73^ and limits researchers’ ability to accurately measure adherence.

MEMS also posed challenges. Despite interviewer instruction, some women initially struggled to open the MEMS cap resulting in husbands helping with “practice openings.” For this reason, electronic monitoring data were capped at 100%. MEMS is limited as an effective monitoring tool for dosing regimens that include separate pills for more than one micronutrient. Thus participants were provided blister-packaged IFA separately, and IFA adherence was not electronically monitored.

Women’s reluctance to take calcium in public may have been exacerbated by the large size of the MEMS cap and instructions to travel with the bottle rather than remove tablets for later dosing. “Pocket dosing” facilitated taking a midday dose but could have resulted in inaccuracies in MEMS data. Additionally, knowing that pill-taking was monitored may have increased adherence for some.

Although regimens were assigned randomly, the group assigned the regimen with only 2 pill-taking events appeared to have lower rates of food insecurity. One would expect that if this had any effect, it would be to increase adherence, given that food insecurity was mentioned as a barrier, but we did not find higher adherence in this group. Thus, the variation in food security does not change our conclusion that reduced pill-taking events did not result in higher adherence.

Finally, this study was conducted in an area recently served by a maternal health intervention that strengthened various services related to pregnancy care and IFA supplementation^41^; motivations, preferences, and adherence to supplementation in other less well-established program areas may reveal different results.

## CONCLUSION

To reduce preeclampsia and anemia, the WHO recommends antenatal calcium and IFA supplementation in populations with low dietary intake. Concurrent antenatal calcium with IFA supplementation was acceptable for most women in this setting, even in the context of negative community perceptions of pill-taking and low IFA use before the study. Women were eager for extensive information about supplementation and motivated by knowledge about health benefits of calcium and IFA. Social support, regular counseling ad-dressing key barriers, and home-based reminders facilitated calcium adherence. Due to the complexity of the prenatal calcium regimen in the WHO guidelines (1.5 g/d in 3 doses with separate IFA administration), we compared adherence to simpler regimens. Calcium adherence did not differ across regimen; thus, a regimen of fewer calcium doses (1 g/d) that women preferred resulted in lower calcium intakes. A regimen of 3 doses of calcium (1.5 g/d) simplified to allow co-consumption with IFA resulted in over 80% of the women consuming 1,000 mg or more daily. This approach is recommended, pending further research to identify the minimal effective dose to reduce preeclampsia risk. Determining the feasibility of integrating calcium into existing antenatal IFA supplementation programs and achieving high levels of adherence requires further implementation research within health systems, but results indicate high acceptance of calcium supplementation in this context.

## Notes

**Peer Reviewed**

**First published online:** August 25, 2020

## References

[1] Bhutta ZA, Das JK, Rizvi A, et al; Lancet Nutrition Interventions Review Group, the Maternal and Child Nutrition Study Group. Evidence-based interventions for improvement of maternal and child nutrition: what can be done and at what cost? Lancet. 2013;382(9890):452–477. 10.1016/S0140-6736(13)60996-4. 23746776

[2] Casey GJ, Sartori D, Horton SE, et al. Weekly iron-folic acid supplementation with regular deworming is cost-effective in preventing anaemia in women of reproductive age in Vietnam. PLoS One. 2011;6(9):e23723. 10.1371/journal.pone.0023723. 21931611 PMC3169551

[3] Alderman H, Behrman J, Hoddinott J. Nutrition, malnutrition and economic growth. In: López-Casasnovas G, Rivera B, Currais L, eds. *Health and Economic Growth: Findings and Policy Implications*. MIT Press; 2005:169–194.

[4] Barker PM, Reid A, Schall MW. A framework for scaling up health interventions: lessons from large-scale improvement initiatives in Africa. Implement Sci. 2015;11(1):12. 10.1186/s13012-016-0374-x. 26821910 PMC4731989

[5] De-Regil LM, Peña-Rosas JP, Flores-Ayala R, del Socorro Jefferds ME. Development and use of the generic WHO/CDC logic model for vitamin and mineral interventions in public health programmes. Public Health Nutr. 2014;17(3):634–639. 10.1017/S1368980013000554. 23507463 PMC4547471

[6] GBD 2015 Maternal Mortality Collaborators. Global, regional, and national levels of maternal mortality, 1990–2015: a systematic analysis for the Global Burden of Disease Study 2015. Lancet. 2016;388(10053):1775–1812. 10.1016/S0140-6736(16)31470-2. 27733286 PMC5224694

[7] World Health Organization (WHO). *WHO Recommendation: Calcium Supplementation During Pregnancy for the Prevention of Pre-eclampsia and Its Complications*. WHO; 2018. Accessed July 14, 2020. https://apps.who.int/iris/bitstream/handle/10665/277235/9789241550451-eng.pdf 30629391

[8] Hofmeyr GJ, Lawrie TA, Atallah ÁN, Torloni MR. Calcium supplementation during pregnancy for preventing hypertensive disorders and related problems. Cochrane Database Syst Rev. 2018;10:CD001059. 10.1002/14651858.CD001059.pub5. 30277579 PMC6517256

[9] Bentley ME, Johnson SL, Wasser H, et al. Formative research methods for designing culturally appropriate, integrated child nutrition and development interventions: an overview. Ann N Y Acad Sci. 2014;1308(1):54–67. 10.1111/nyas.12290. 24673167 PMC4269231

[10] World Health Organization (WHO). *Guideline: Daily Iron and Folic Acid Supplementation in Pregnant Women*. WHO; 2012. Accessed July 14, 2020. https://apps.who.int/iris/bitstream/handle/10665/77770/9789241501996_eng.pdf 23586119

[11] Siekmans K, Roche M, Kung’u JK, Desrochers RE, De-Regil LM. Barriers and enablers for iron folic acid (IFA) supplementation in pregnant women. Matern Child Nutr. 2018;14(Suppl 5):e12532. 10.1111/mcn.12532. 29271115 PMC6865983

[12] Ba DM, Ssentongo P, Kjerulff KH, et al. Adherence to iron supplementation in 22 sub-Saharan African countries and associated factors among pregnant women: a large population-based study. Curr Dev Nutr. 2019;3(12):nzz120. 10.1093/cdn/nzz120. 31777771 PMC6867960

[13] Desta M, Kassie B, Chanie H, et al. Adherence of iron and folic acid supplementation and determinants among pregnant women in Ethiopia: a systematic review and meta-analysis. Reprod Health. 2019;16(1):182. 10.1186/s12978-019-0848-9. 31864397 PMC6925441

[14] Molla T, Guadu T, Muhammad EA, Hunegnaw MT. Factors associated with adherence to iron folate supplementation among pregnant women in West Dembia district, northwest Ethiopia: a cross sectional study. BMC Res Notes. 2019;12(1):6. 10.1186/s13104-019-4045-2. 30612583 PMC6322306

[15] Gebremichael TG, Welesamuel TG. Adherence to iron-folic acid supplement and associated factors among antenatal care attending pregnant mothers in governmental health institutions of Adwa town, Tigray, Ethiopia: cross-sectional study. PLoS One. 2020;15(1):e0227090. 10.1371/journal.pone.0227090. 31910215 PMC6946125

[16] Yip R. Iron supplementation during pregnancy: is it effective? Am J Clin Nutr. 1996;63(6):853–855. 10.1093/ajcn/63.6.853. 8644677

[17] Galloway R, Dusch E, Elder L, et al. Women’s perceptions of iron deficiency and anemia prevention and control in eight developing countries. Soc Sci Med. 2002;55(4):529–544. 10.1016/S0277-9536(01)00185-X. 12188461

[18] Garcia-Casal MN, Estevez D, De-Regil LM. Multiple micronutrient supplements in pregnancy: Implementation considerations for integration as part of quality services in routine antenatal care. Objectives, results, and conclusions of the meeting. Matern Child Nutr. 2018;14(5)(Suppl 5):e12704. 10.1111/mcn.12704. 30585705 PMC6866095

[19] Stoltzfus RJ. Iron interventions for women and children in low-income countries. J Nutr. 2011;141(4):756S–762S. 10.3945/jn.110.128793. 21367936

[20] Sanghvi TG, Harvey PWJ, Wainwright E. Maternal iron-folic acid supplementation programs: evidence of impact and implementation. Food Nutr Bull. 2010;31(2_Suppl):S100–S107. 10.1177/15648265100312S202. 20715594

[21] Birhanu Z, Chapleau GM, Ortolano SE, Mamo G, Martin SL, Dickin KL. Ethiopian women’s perspectives on antenatal care and iron-folic acid supplementation: insights for translating global antenatal calcium guidelines into practice. Matern Child Nutr. 2018;14(1)(Suppl 1):e12424. 10.1111/mcn.12424. 29493899 PMC6866194

[22] Martin SL, Seim GL, Wawire S, Chapleau GM, Young SL, Dickin KL. Translating formative research findings into a behaviour change strategy to promote antenatal calcium and iron and folic acid supplementation in western Kenya. Matern Child Nutr. 2017;13(1):mcn. 12233. 10.1111/mcn.12233. 26898417 PMC6866120

[23] Baxter JAB, Roth DE, Al Mahmud A, Ahmed T, Islam M, Zlotkin SH. Tablets are preferred and more acceptable than powdered prenatal calcium supplements among pregnant women in Dhaka, Bangladesh. J Nutr. 2014;144(7):1106–1112. 10.3945/jn.113.188524. 24759933

[24] Joshi M, Gumashta R. Weekly iron folate supplementation in adolescent girls—an effective nutritional measure for the management of iron deficiency anaemia. Glob J Health Sci. 2013;5(3):188–194. 10.5539/gjhs.v5n3p188. 23618489 PMC4776831

[25] Omotayo MO, Dickin KL, Pelletier DL, Martin SL, Kung’u JK, Stoltzfus RJ. Feasibility of integrating calcium and iron-folate supplementation to prevent preeclampsia and anemia in pregnancy in primary healthcare facilities in Kenya. Matern Child Nutr. 2018;14(1)(Suppl 1):e12437. 10.1111/mcn.12437. 29493897 PMC6866141

[26] Nguyen PH, Sanghvi T, Kim SS, et al. Factors influencing maternal nutrition practices in a large scale maternal, newborn and child health program in Bangladesh. PLoS One. 2017;12(7):e0179873. 10.1371/journal.pone.0179873. 28692689 PMC5503174

[27] Thapa K, Sanghvi H, Rawlins B, et al. Coverage, compliance, acceptability and feasibility of a program to prevent pre-eclampsia and eclampsia through calcium supplementation for pregnant women: an operations research study in one district of Nepal. BMC Pregnancy Childbirth. 2016;16(1):241. 10.1186/s12884-016-1033-6. 27553004 PMC4995763

[28] Dickin KL, Seim G. Adapting the trials of improved practices (TIPs) approach to explore the acceptability and feasibility of nutrition and parenting recommendations: what works for low-income families? Matern Child Nutr. 2015;11(4):897–914. 10.1111/mcn.12078. 24028083 PMC6860192

[29] Dickin K, Griffiths M, Piwoz E. *Designing by Dialogue: A Program Planners’ Guide to Consultative Research for Improving Young Child Feeding*. Academy for Educational Development; 1997.

[30] Srivastava K, Arora A, Kataria A, Cappelleri JC, Sadosky A, Peterson AM. Impact of reducing dosing frequency on adherence to oral therapies: a literature review and meta-analysis. Patient Prefer Adherence. 2013;7:419–434. 10.2147/ppa.s44646. 23737662 PMC3669002

[31] Ingersoll KS, Cohen J. The impact of medication regimen factors on adherence to chronic treatment: a review of literature. J Behav Med. 2008;31(3):213–224. 10.1007/s10865-007-9147-y. 18202907 PMC2868342

[32] Osterberg L, Blaschke T. Adherence to medication. N Engl J Med. 2005;353(5):487–497. 10.1056/NEJMra050100. 16079372

[33] Claxton AJ, Cramer J, Pierce C. A systematic review of the associations between dose regimens and medication compliance. Clin Ther. 2001;23(8):1296–1310. 10.1016/S0149-2918(01)80109-0. 11558866

[34] Lönnerdal B. Calcium and iron absorption—mechanisms and public health relevance. Int J Vitam Nutr Res. 2010;80(45):293–299. 10.1024/0300-9831/a000036. 21462112

[35] Omotayo MO, Dickin KL, O’Brien KO, Neufeld LM, De Regil LM, Stoltzfus RJ. Calcium supplementation to prevent preeclampsia: translating guidelines into practice in low-income countries. Adv Nutr. 2016;7(2):275–278. 10.3945/an.115.010736. 26980810 PMC4785477

[36] Hofmeyr GJ, Belizán JM, von Dadelszen P; Calcium and Pre-eclampsia (CAP) Study Group. Low-dose calcium supplementation for preventing pre-eclampsia: a systematic review and commentary. BJOG. 2014;121(8):951–957. 10.1111/1471-0528.12613. 24621141 PMC4282055

[37] Government of the Federal Democratic Republic of Ethiopia. *National Nutrition Programme June 2013–2015*. Addis Ababa, Ethiopia. Accessed July 14, 2020. https://extranet.who.int/nutrition/gina/sites/default/files/ETH%202013%20National%20Nutrition%20Programme.pdf

[38] Federal Democratic Republic of Ethiopia. Central Statistical Agency (CSA); ICF. *Ethiopia Demographic and Health Survey 2016*. CSA and ICF; July 2017. Accessed July 14, 2020. https://dhsprogram.com/pubs/pdf/FR328/FR328.pdf

[39] Gaym A, Bailey P, Pearson L, Admasu K, Gebrehiwot Y; Ethiopian National EmONC Assessment Team. Disease burden due to pre-eclampsia/eclampsia and the Ethiopian health system’s response. Int J Gynaecol Obstet. 2011;115(1):112–116. 10.1016/j.ijgo.2011.07.012. 21849170

[40] Smith JM, Currie S, Cannon T, Armbruster D, Perri J. Are national policies and programs for prevention and management of postpartum hemorrhage and preeclampsia adequate? A key informant survey in 37 countries. Glob Health Sci Pract. 2014;2(3):275–284. 10.9745/GHSP-D-14-00034. 25276587 PMC4168639

[41] Nutrition International. Why Iron. Accessed July 14, 2020. http://www.micronutrient.org/what-we-do/by-micronutrient/iron/

[42] Patton MQ. *Qualitative Research & Evaluation Methods: Integrating Theory and Practice*. Sage Publications; 2014.

[43] Martin SL, Omotayo MO, Pelto GH, Chapleau GM, Stoltzfus RJ, Dickin KL. Adherence-specific social support enhances adherence to calcium supplementation regimens among pregnant women. J Nutr. 2017;147(4):688–696. 10.3945/jn.116.242503. 28250195

[44] Proctor E, Silmere H, Raghavan R, et al. Outcomes for implementation research: conceptual distinctions, measurement challenges, and research agenda. Adm Policy Ment Health. 2011;38(2):65–76. 10.1007/s10488-010-0319-7. 20957426 PMC3068522

[45] Corbin JM, Strauss A. Grounded theory research: procedures, canons, and evaluative criteria. Qual Sociol. 1990;13(1):3–21. 10.1007/BF00988593

[46] MacQueen KM, McLellan E, Kay K, Milstein B. Codebook development for team-based qualitative analysis. CAM Journal. 1998;10(12):31–36. 10.1177/1525822X980100020301

[47] Ritchie J, Lewis J. *Qualitative Research Practice: A Guide for Social Science Students and Researchers*. Sage Publications Ltd; 2014.

[48] Thurmond VA. The point of triangulation. J Nurs Scholarsh. 2001;33(3):253–258. 10.1111/j.1547-5069.2001.00253.x. 11552552

[49] Kimchi J, Polivka B, Stevenson JS. Triangulation: operational definitions. Nurs Res. 1991;40(6):364–366. 10.1097/00006199-199111000-00009. 1956817

[50] Institute of Medicine (US) Committee to Review Dietary Reference Intakes for Vitamin D and Calcium; Ross AC, Taylor CL, Yaktine AL, et al. *Dietary Reference Intakes for Calcium and Vitamin D*. National Academies Press; 2011. Accessed July 28. 2020. https://www.ncbi.nlm.nih.gov/books/NBK56050 21796828

[51] Berhe AK, Kassa GM, Fekadu GA, Muche AA. Prevalence of hypertensive disorders of pregnancy in Ethiopia: a systemic review and meta-analysis. BMC Pregnancy Childbirth. 2018;18(1):34. 10.1186/s12884-018-1667-7. 29347927 PMC5774029

[52] Berhan Y, Berhan A. Causes of maternal mortality in Ethiopia: a significant decline in abortion related death. Ethiop J Health Sci. 2014;24 Suppl(0 Suppl):15–28. 10.4314/ejhs.v24i0.3S. 25489180 PMC4249203

[53] Omotayo MO, Martin SL, Stoltzfus RJ, Ortolano SE, Mwanga E, Dickin KL. With adaptation, the WHO guidelines on calcium supplementation for prevention of pre‐eclampsia are adopted by pregnant women. Matern Child Nutr. 2018;14(2):e12521. 10.1111/mcn.12521. 29193667 PMC6865867

[54] Kassa ZY, Awraris T, Daba AK, Tenaw Z. Compliance with iron folic acid and associated factors among pregnant women through pill count in Hawassa city, South Ethiopia: a community based cross-sectional study. Reprod Health. 2019;16(1):14. 10.1186/s12978-019-0679-8. 30736812 PMC6367743

[55] Taye B, Abeje G, Mekonen A. Factors associated with compliance of prenatal iron folate supplementation among women in Mecha district, Western Amhara: a cross-sectional study. Pan Afr Med J. 2015;20:43. 10.11604/pamj.2015.20.43.4894. 26090001 PMC4449983

[56] Arega Sadore A, Abebe Gebretsadik L, Aman Hussen M. Compliance with iron-folate supplement and associated factors among antenatal care attendant mothers in Misha District, South Ethiopia: community based cross-sectional study. J Environ Public Health. 2015;2015: 781973. 10.1155/2015/781973. 26839573 PMC4709613

[57] Asfaw S, Morankar S, Abera M, et al. Talking health: trusted health messengers and effective ways of delivering health messages for rural mothers in Southwest Ethiopia. Arch Public Health. 2019;77(1):8. 10.1186/s13690-019-0334-4. 30828451 PMC6383212

[58] Khanam F, Hossain B, Mistry SK, et al. The association between daily 500 mg calcium supplementation and lower pregnancy-induced hypertension risk in Bangladesh. BMC Pregnancy Childbirth. 2018;18(1):406. 10.1186/s12884-018-2046-0. 30332997 PMC6192122

[59] Demonstrating non‐inferiority of lower dose calcium supplement-ation during pregnancy for reducing preeclampsia and neonatal outcomes. ClinicalTrials.gov identifier: NCT03350516. Updated November 22, 2017. Accessed February 4, 2020. https://clinicaltrials.gov/ct2/show/record/NCT03350516?view=record

[60] Shivalli S, Srivastava RK, Singh GP. Trials of improved practices (TIPs) to enhance the dietary and iron-folate intake during pregnancy—a quasi experimental study among rural pregnant women of Varanasi, India. PLoS One. 2015;10(9):e0137735. 10.1371/journal.pone.0137735. 26367775 PMC4569533

[61] Federal Democratic Republic of Ethiopia Ministry of Health (MOH). *Guidelines for the Prevention and Control of Micronutrient Deficiencies in Ethiopia*. MOH; 2016:47–50.

[62] Kavle JA, Landry M. Community-based distribution of iron–folic acid supplementation in low- and middle-income countries: a review of evidence and programme implications. Public Health Nutr. 2018;21(2):346–354. 10.1017/S1368980017002828. 29061205 PMC10261080

[63] Cormick G, Betrán AP, Romero IB, et al. Global inequities in dietary calcium intake during pregnancy: a systematic review and meta‐analysis. BJOG. 2019;126(4):444–456. 10.1111/1471-0528.15512. 30347499 PMC6518872

[64] Tesfaye B, Sinclair K, Wuehler SE, Moges T, De-Regil LM, Dickin KL. Applying international guidelines for calcium supplementation to prevent pre-eclampsia: simulation of recommended dosages suggests risk of excess intake in Ethiopia. Public Health Nutr. 2019;3:531–541. 10.1017/S1368980018002562. 30319089 PMC10260678

[65] Mohammed SH, Taye H, Sissay TA, Larijani B, Esmaillzadeh A. Teff consumption and anemia in pregnant Ethiopian women: a case–control study. Eur J Nutr. 2019;58(5):2011–2018. 10.1007/s00394-018-1759-1. 29936535

[66] Abay A, Yalew HW, Tariku A, Gebeye E. Determinants of prenatal anemia in Ethiopia. Arch Public Health. 2017;75(1):51. 10.1186/s13690-017-0215-7. 29142745 PMC5674228

[67] Abriha A, Yesuf M, Wassie M. Prevalence and associated factors of anemia among pregnant women of Mekelle town: a cross sectional study. BMC Res Notes. 2014;7(1):888. 10.1186/1756-0500-7-888. 25487251 PMC4295569

[68] Torjusen H, Brantsaeter AL, Haugen M, et al. Reduced risk of pre-eclampsia with organic vegetable consumption: results from the prospective Norwegian Mother and Child Cohort Study. BMJ Open. 2014;4(9):e006143. 10.1136/bmjopen-2014-006143. 25208850 PMC4160835

[69] Longo-Mbenza B, Tshimanga BK, Buassa-bu-Tsumbu B, Kabangu JR. Diets rich in vegetables and physical activity are associated with a decreased risk of pregnancy induced hypertension among rural women from Kimpese, DR Congo. Niger J Med. 2008;17(3):265–269. 10.4314/njm.v17i3.37393. 18788250

[70] Agrawal S, Fledderjohann J, Vellakkal S, Stuckler D. Adequately diversified dietary intake and iron and folic acid supplementation during pregnancy is associated with reduced occurrence of symptoms suggestive of pre-eclampsia or eclampsia in Indian women. PLoS One. 2015;10(3):e0119120. 10.1371/journal.pone.0119120. 25785774 PMC4364955

[71] Omotayo MO, Dickin KL, Pelletier DL, Mwanga EO, Kung’u JK, Stoltzfus RJ. A simplified regimen compared with WHO guidelines decreases antenatal calcium supplement intake for prevention of preeclampsia in a cluster-randomized noninferiority trial in rural Kenya. J Nutr. 2017;147(10):1986–1991. 10.3945/jn.117.251926. 28878035

[72] Krezanoski PJ, Bangsberg DR, Tsai AC. Quantifying bias in measuring insecticide-treated bednet use: meta-analysis of self-reported vs objectively measured adherence. J Glob Health. 2018;8(1):010411. 10.7189/jogh.08.010411. 29619211 PMC5878861

[73] Teshome EM, Oriaro VS, Andango PEA, Prentice AM, Verhoef H. Adherence to home fortification with micronutrient powders in Kenyan pre-school children: self-reporting and sachet counts compared to an electronic monitoring device. BMC Public Health. 2018;18(1):205. 10.1186/s12889-018-5097-2. 29391008 PMC5796300

